# Monitoring the impact of a national school based deworming programme on soil-transmitted helminths in Kenya: the first three years, 2012 – 2014

**DOI:** 10.1186/s13071-016-1679-y

**Published:** 2016-07-25

**Authors:** Collins Okoyo, Birgit Nikolay, Jimmy Kihara, Elses Simiyu, Joshua V. Garn, Mathew C. Freeman, Mariam T. Mwanje, Dunstan A. Mukoko, Simon J. Brooker, Rachel L. Pullan, Sammy M. Njenga, Charles S. Mwandawiro

**Affiliations:** 1 Eastern and Southern Africa Centre of International Parasite Control, Kenya Medical Research Institute (KEMRI), Nairobi, Kenya; 2 Faculty of Infectious and Tropical Diseases, London School of Hygiene and Tropical Medicine, Keppel Street, London, WC1E 7HT UK; 3 Department of Environmental Health, Rollins School of Public Health, Emory University, Atlanta, GA USA; 4 Division of Vector-borne and Neglected Tropical Diseases, Ministry of Public Health and Sanitation, Nairobi, P.O. Box 19982-00202 Kenya

**Keywords:** Hookworms, *Ascaris lumbricoides*, *Trichuris trichiura*, School-based deworming

## Abstract

**Background:**

In 2012, the Kenyan Ministries of Health and of Education began a programme to deworm all school-age children living in areas at high risk of soil-transmitted helminths (STH) and schistosome infections. The impact of this school-based mass drug administration (MDA) programme in Kenya is monitored by the Kenya Medical Research Institute (KEMRI) as part of a five-year (2012–2017) study. This article focuses on the impact of MDA on STH infections and presents the overall achieved reductions from baseline to mid-term, as well as yearly patterns of reductions and subsequent re-infections per school community.

**Methods:**

The study involved a series of pre- and post-intervention, repeat cross-sectional surveys in a representative, stratified, two-stage sample of schools across Kenya. The programme contained two tiers of monitoring; a national baseline and mid-term survey including 200 schools, and surveys conducted among 60 schools pre- and post-intervention. Stool samples were collected from randomly selected school children and tested for helminth infections using Kato-Katz technique. The prevalence and mean intensity of each helminth species were calculated at the school and county levels and 95 % confidence intervals (CIs) were obtained by binomial and negative binomial regression, respectively, taking into account clustering by schools.

**Results:**

The overall prevalence of STH infection at baseline was 32.3 % (hookworms: 15.4 %; *Ascaris lumbricoides*: 18.1 %; and *Trichuris trichiura*: 6.7 %). After two rounds of MDA, the overall prevalence of STH had reduced to 16.4 % (hookworms: 2.3 %; *A. lumbricoides*: 11.9 %; and *T. trichiura*: 4.5 %). The relative reductions of moderate to heavy intensity of infections were 33.7 % (STH combined), 77.3 % (hookworms) and 33.9 % (*A. lumbricoides*). For *T. trichiura,* however, moderate to heavy intensity of infections increased non-significantly by 18.0 % from baseline to mid-term survey.

**Conclusion:**

The school-based deworming programme has substantially reduced STH infections, but because of ongoing transmission additional strategies may be required to achieve a sustained interruption of transmission.

**Electronic supplementary material:**

The online version of this article (doi:10.1186/s13071-016-1679-y) contains supplementary material, which is available to authorized users.

## Background

Helminth infections are a major public health problem and cause mal-nutrition and cognitive impairment [1–4], with school children typically experiencing the heaviest burden of disease [5]. Soil-transmitted helminths (STH: *Ascaris lumbricoides*, *Trichuris trichiura*, and the hookworms *Necator americanus* and *Ancylostoma duodenale*) are among the diseases classified by the World Health Organization as neglected tropical diseases (NTDs) [6]. STH are endemic in nearly 166 countries globally [7] with majority of them now implementing mass drug administration (MDA) programmes, either through school-based deworming (SBD) or lymphatic filariasis control programmes [8, 9].

STH affects more than two billion people worldwide. Recent estimates suggest that *A. lumbricoides* infects 1.2 billion people, *T. trichiura* 795 million, and hookworms (*Ancylostoma duodenale* and *Necator americanus*) 740 million [10]. The greatest numbers of STH infections occur in sub-Saharan Africa, the Americas, China and East Asia. Warm climates and adequate moisture are essential for the hatching or embryonation of STH eggs in the environment or development of larvae.

Infection is associated with ingestion of eggs from contaminated soil (*A. lumbricoides* and *T. trichiura*) or by active penetration of the skin by larvae in the soil (hookworms) [11]. People infected with STHs produce a wide range of symptoms that include intestinal manifestations (diarrhoea, abdominal pain), general malaise and weakness that may affect working and learning capacities, and impaired physical growth. Hookworms cause chronic intestinal blood loss that result in anaemia. An important epidemiological feature is their highly aggregated distribution; the majority of patients harbour low intensity of infections, while only few individuals harbour very heavy infections [12].

Although school-based deworming has many benefits for treated children, it does not prevent re-infections, which can occur rapidly after treatment, particularly for *A. lumbricoides* and *T. trichiura*. Hence, there is a need for frequent and consistent MDA to maximise the benefit of preventive chemotherapy [13].

In 2012, the Kenyan Ministries of Health and Education began a school-based deworming program in 66 districts (now sub-counties) identified as having a high prevalence of STH and schistosome infections in four regions (Western, Nyanza, Rift Valley and Coast). According to the Kenyan National School Health Policy, treatment is administered to all school-age children, including those out of school, based on the prevalence and intensity of STH and schistosome infections in the area in order to reduce infection. The goal of the school-based deworming programme is to reduce the prevalence of moderate to heavy infections to < 1 % such that the infections are no longer of public health importance [14]. The programme is embedded within the National Multi-Year Strategic Plan for the Control of Neglected Tropical Diseases, which was launched in 2011, and lays the basis for a comprehensive strategy for the integration of neglected tropical disease control efforts [14].

The impact of the Kenyan school-based deworming programme on STH and schistosome infections is being monitored through to 2017 with specific objectives outlined elsewhere [15], and includes pre-post intervention and repeated cross-sectional surveys as outlined in Additional file 1: Figure S1. Our analysis herein presents the findings on STH infections in 200 schools surveyed for baseline (2012) and mid-term (2014) assessment and in 60 schools surveyed yearly from 2012 to 2015 in the above mentioned regions. The results for schistosome infections are not reported due to the inconsistency in the delivery of praziquantel treatment caused by logistical challenges.

The specific aims of this study were to (i) determine the overall achieved reductions in infections from baseline to mid-term; (ii) determine the yearly patterns of treatment impact and re-infections per school community; and (iii) determine the impact on infections of high intensity (moderate or heavy infections).

## Methods

### Study design

Monitoring and evaluation (M&E) of the STH programme includes a series of pre- and post-intervention, repeat cross-sectional surveys in a representative, stratified, two-stage sample of schools across Kenya. Sub-county stratification was based on both geography and anticipated infection prevalence. There are two tiers of monitoring: (i) a national baseline, mid-term (after two MDA rounds) and end-term survey (after four MDA rounds) including 200 schools in 20 sub-counties from 16 counties, that aimed to establish an accurate national measurement of infection levels and monitor long term changes in worm infection levels; and (ii) surveys conducted in 60 of the 200 schools before and 3–5 weeks after the deworming activity (pre-post surveys) to evaluate reductions in infections that can be directly attributed to programme implementation (Additional file 1: Figure S1) [15].

A sample size of 200 schools for the baseline, mid-term and end-term assessment was determined to be adequate to detect a 5 % change in prevalence across years, assuming a power of *β* = 0.8 and test size *α* = 0.05, and considering the anticipated variance in prevalence [15].

The 200 schools were selected randomly from 66 sub-counties (formerly districts) based on the geographical distribution of the population and STH endemicity. Available data and predictive maps [16, 17] indicated endemicity of STH in 66 sub-counties. From these sub-counties, grouped into infection level strata, 20 sub-counties from 16 counties were randomly selected in the first sampling stage, with the number of sub-counties per region proportional to population: six sub-counties from Western region, three from Rift Valley, five from Coast and six from Nyanza. At the second sampling stage, primary schools were randomly selected from within the chosen 20 sub-counties (Table 1). A detailed description of this M&E programme design is provided together with the baseline assessment by Mwandawiro et al. [15].

**Table 1:** Number of schools (children) sampled in each region for each tier of monitoring

Region	Baseline, mid-term & endterm	Pre-post	Totals
Western	42 (4536)	18 (1944)	60 (6480)
Rift Valley	20 (2160)	9 (972)	29 (3132)
Coast	37 (3996)	15 (1620)	52 (5616)
Nyanza	41 (4428)	18 (1944)	59 (6372)
Totals	140 (15,120)	60 (6480)	200 (21,600)

### Data collection

Baseline surveys in 200 schools were conducted between January and April 2012 and year 1 (Y1) post-MDA surveys were conducted in 60 schools between September 2012 and May 2013. In year 2 (Y2), the pre-MDA surveys took place between May 2013 and February 2014 and the post-MDA surveys took place between July 2013 and April 2014. Mid-term surveys in year 3 (Y3) were carried out between March 2014 and February 2015, followed by post-MDA surveys in October 2014 and July 2015. In each school, 18 children (9 girls and 9 boys) were sampled randomly from each of the six classes; one Early Childhood Development (ECD) class and classes 2–6 using random number tables, for a total of approximately 108 children per school. Information was captured electronically during the interviews using Open Data Kit (ODK) for android-based smartphones [18] that stored, provisionally checked and later transmitted data to KEMRI headquarters in Nairobi.

### Survey procedures

Selected schools were visited one week prior to the survey date to explain the purpose of the survey to head teachers and school committees, and permission was sought at the school-level. Parental consent from parents/guardians of children in ECD and classes 2–6 was based on passive, opt-out consent rather than written opt-in consent. Selected children were asked to provide stool samples which were examined in duplicate for the presence of STH eggs by two different technicians using the Kato-Katz method; any discrepancies were resolved by a third senior technician. Ten percent of all samples were re-examined by a senior technician. The presence of each infection was recorded as eggs per gram of stool. Within the national deworming programme, all participants received treatment with albendazole (400 mg) for STH infection according to World Health Organization (WHO) guidelines [19].

### Statistical analysis

The overall prevalence of each helminth species and STH combined was calculated at the school and county level and the 95 % confidence intervals (CIs) were obtained by binomial regression taking into account school clusters. Mean egg counts were expressed as arithmetic mean eggs per gram of feaces (epg) and since the distribution of egg counts was overdispersed, 95 % CIs were obtained using negative binomial regression models taking into account school clusters. Infection intensities were classified into light, moderate and heavy infections according to WHO guidelines (Additional file 1: Table S1) and the prevalence of each infection class together with 95 % CIs were obtained using binomial regression adjusting for clustering by schools. The relative reductions in prevalence and intensity of each STH species and light to heavy infections from Y1 baseline to Y3 Pre-MDA were calculated using multivariable mixed effects models with random intercepts for schools and counties and *P*-values were obtained using Wald test. We also estimated the absolute reductions in prevalence of each STH species using multivariable mixed effects models. All statistical analyses were carried out using STATA version 14.0 (STATA Corporation, College Station, TX, USA). Graphs were developed using the *ggplot* package implemented in R [20]. School locations were mapped using ArcGIS Desktop version 10.2.2 software (Environmental Systems Research Institute Inc., Redlands, CA, USA). Hotspot analysis for STH infections at the school level was carried out using the ArcGIS’s cluster and outlier analysis tool, a school was identified as a significant hotspot for any of the STH based on the Anselin Local Moran’s I statistic [21].

## Results

In total, 199 schools across 16 counties in Western, Nyanza, Rift valley and Coast regions were included in the Y1 baseline and Y3 mid-term assessment, 59 schools were included in the follow-up annual surveys. One school in Bungoma County was replaced after the baseline survey and was therefore excluded together with the replacement school to allow for comparability between baseline and follow-up surveys. The number of schools and children surveyed per county at each time point is shown in Table 2. Child age was obtained for 66,888 children (99.4 %) and ranged from 2 to 19 years with a mean age of 10 years (standard deviation 2.7 years). Information on sex was recorded for 66,976 children (99.6 %); of these 50.2 % were male. Children absent on the day of the survey were not included in the study.

**Table 2:** Number of schools (children) examined in each survey

County	Year 1 (2012)		Year 2 (2013)		Year 3 (2014)	
	Baseline	Post-MDA	Pre-MDA	Post-MDA	Mid-term	Post-MDA
Bomet	12 (1296)	3 (324)	3 (313)	3 (319)	12 (1298)	3 (313)
Bungoma^a^	9 (968)	2 (216)	2 (215)	2 (216)	9 (935)	2 (203)
Busia	18 (1942)	6 (648)	6 (641)	6 (643)	18 (1927)	6 (647)
Homa bay	24 (2590)	6 (642)	6 (646)	6 (634)	24 (2483)	6 (631)
Kakamega	20 (2152)	6 (648)	6 (641)	6 (644)	20 (2086)	6 (608)
Kericho	12 (1292)	3 (324)	3 (312)	3 (279)	12 (1297)	3 (295)
Kilifi	10 (1080)	3 (316)	3 (324)	3 (324)	10 (1069)	3 (315)
Kisii	12 (1296)	3 (324)	3 (320)	3 (318)	12 (1265)	3 (317)
Kisumu	10 (1078)	3 (324)	3 (295)	3 (313)	10 (1032)	3 (323)
Kwale	18 (1940)	6 (642)	6 (648)	6 (648)	18 (1884)	6 (563)
Migori	8 (864)	3 (226)	3 (323)	3 (314)	8 (863)	3 (314)
Mombasa	8 (852)	3 (313)	3 (324)	3 (324)	8 (844)	3 (311)
Narok	10 (1070)	3 (324)	3 (322)	3 (274)	10 (1062)	3 (311)
Nyamira	10 (1080)	3 (324)	3 (321)	3 (320)	10 (1073)	3 (313)
Taita Taveta	10 (1072)	3 (311)	3 (318)	3 (324)	10 (1068)	3 (322)
Vihiga	8 (860)	3 (324)	3 (319)	3 (320)	8 (825)	3 (311)
Total	199 (21,432)	59 (6230)	59 (6282)	59 (6214)	199 (21,011)	59 (6097)

^a^one school was replaced in Bungoma County and was therefore excluded together with the replacement school

### Reduction of infections from baseline to mid-term assessment

In the baseline survey, the overall prevalence of combined STH infection in the 199 schools was 32.3 % (95 % CI: 30.0–34.8). *A. lumbricoides* was the most common STH species, with 18.1 % (95 % CI: 15.8–20.7) of children infected, followed by hookworms with 15.4 % (95 % CI: 13.6–17.6) and *T. trichiura* with 6.7 % (95 % CI: 5.4–8.2). In the mid-term survey, the combined STH prevalence was reduced to 16.4 % (95 % CI: 14.4–18.6); with *A. lumbricoides* reduced to 11.9 % (95 % CI: 10.2–13.9), hookworms to 2.3 % (95 % CI: 1.8–3.0) and *T. trichiura* to 4.5 % (95 % CI: 3.4–6.0). These translated to a relative reduction in the prevalence of combined STH of 49.3 % (Wald test: *Z* = -13.69, *P* < 0.001), with species-specific relative reductions of 85.0 % (Wald test: *Z* = -14.65, *P* < 0.001) for hookworm, 34.0 % (Wald test: *Z* = -8.81, *P* < 0.001) for *A. lumbricoides* and 32.3 % (Wald test: *Z* = -3.91, *P* < 0.001) for *T. trichiura*.

The overall mean intensity of infection at baseline was 63 epg (95 % CI: 50–81) for hookworms, 1659 epg (95 % CI: 1378–1998) for *A. lumbricoides*, and 33 epg (95 % CI: 11–105) for *T. trichiura*. At mid-term, the mean intensity of infection was 8 epg (95 % CI: 5–14) for hookworms, 960 epg (95 % CI: 801–1151) for *A. lumbricoides* and 17 epg (95 % CI: 11–26) for *T. trichiuria* (Table 3). These translated to a relative reduction in mean intensity of 87.1 % (Wald test: *Z* = -6.60, *P* < 0.001) for hookworm, 42.1 % (Wald test: *Z* = -7.98, *P* < 0.001) for *A. lumbricoides* and 49.5 % (Wald test: *Z* = -1.11, *P* = 0.267) for *T. trichiura*.

**Table 3:** Overall prevalence (P, %), mean intensity (epg), relative reductions % (Wald test: *Z*-statistic, *P*-value) and absolute reductions % (Wald test: *Z*-statistic, *P*-value) of STHs: Baseline and mid-term

Infection	Y1 baseline	Y3 mid-term	PRR %	ARP %
Prevalence	P % (95 % CI)	P% (95 % CI)	(*Z*-statistic, *P*-value)	(*Z*-statistic, *P*-value)
STH combined	32.3 (30.0–34.8)	16.4 (14.4–18.6)	49.3 (*Z* = -13.69, *P* < 0.001)	15.9 (*Z* = -16.95, *P* < 0.001)
Hookworms	15.4 (13.6–17.6)	2.3 (1.8–3.0)	85.0 (*Z* = -14.65, *P* < 0.001)	13.1 (*Z* = -13.92, *P* < 0.001)
*A. lumbricoides*	18.1 (15.8–20.7)	11.9 (10.2–13.9)	34.0 (*Z* = -8.81, *P* < 0.001)	6.1 (*Z* = -8.15, *P* < 0.001)
*T. trichiura*	6.7 (5.4–8.2)	4.5 (3.4–6.0)	32.3 (*Z* = -3.91, *P* < 0.001)	2.1 (*Z* = -4.36, *P* < 0.001)
Mean intensity	epg (95 % CI)	epg (95 % CI)	IRR % (*Z*-statistic, *P*-value)	
Hookworms	63 (50–81)	8 (5–14)	87.1 (*Z* = -6.60, *P* < 0.001)	–
*A. lumbricoides*	1659 (1378–1998)	960 (801–1151)	42.1 (*Z* = -7.98, *P* < 0.001)	–
*T. trichiura*	33 (11–105)	17 (11–26)	49.5 (*Z* = -1.11, *P* = 0.267)	–

*Abbreviations*: *PRR* relative reduction in prevalence, *IRR* relative reduction in mean intensity, *ARP* absolute reduction in prevalence

Additional file 1: Table S2 presents the baseline and mid-term prevalence of each STH species and relative reductions by county. At baseline, hookworm prevalence was between 20 and 50 % in Bungoma, Busia, Kakamega, Migori, Kilifi and Kwale counties; *A. lumbricoides* prevalence was between 20 and 50 % in Bomet, Kericho, Kisii, Narok, Nyamira, Bungoma, Kakamega and Vihiga counties; and *T. trichiura* prevalence was between 20 and 50 % in Narok County only. Only two counties (Narok and Vihiga) had prevalence of any STH above 50 %. Relative reductions in prevalences were higher for hookworms with seven counties recording > 90 % reductions; for *A. lumbricoides* only two counties (Mombasa and Taita Taveta) recorded > 90 % reductions while Kilifi showed 81.6 % reduction. Similarly, relative reductions for *T. trichiura* were greater than 90 % in only one county. Baseline and mid-term mean intensity of each STH species and relative reductions by county are presented in (Additional file 1: Table S3).

**Fig. 1:**
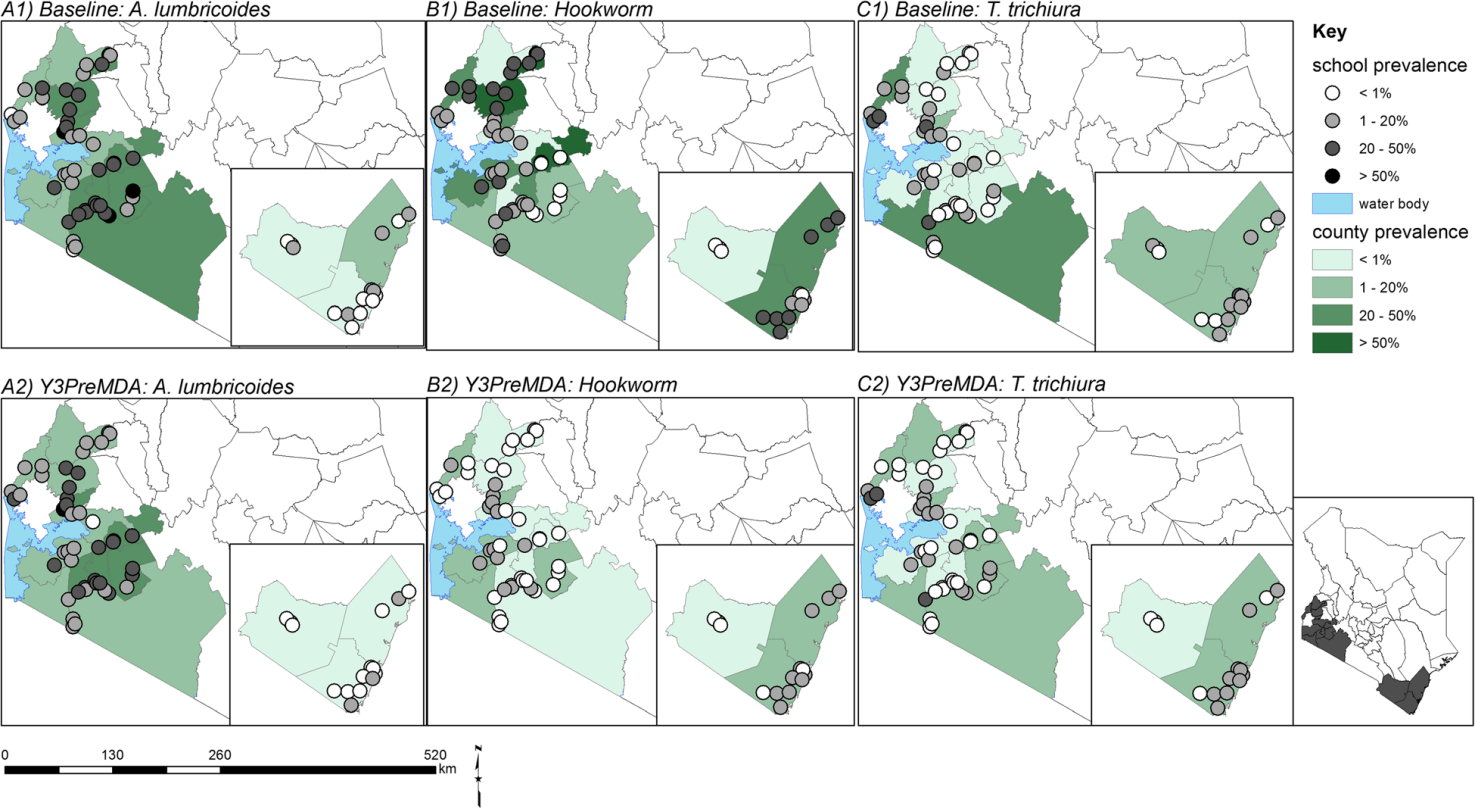
Observed geographical distribution of infection prevalence (%)

**Fig. 2:**
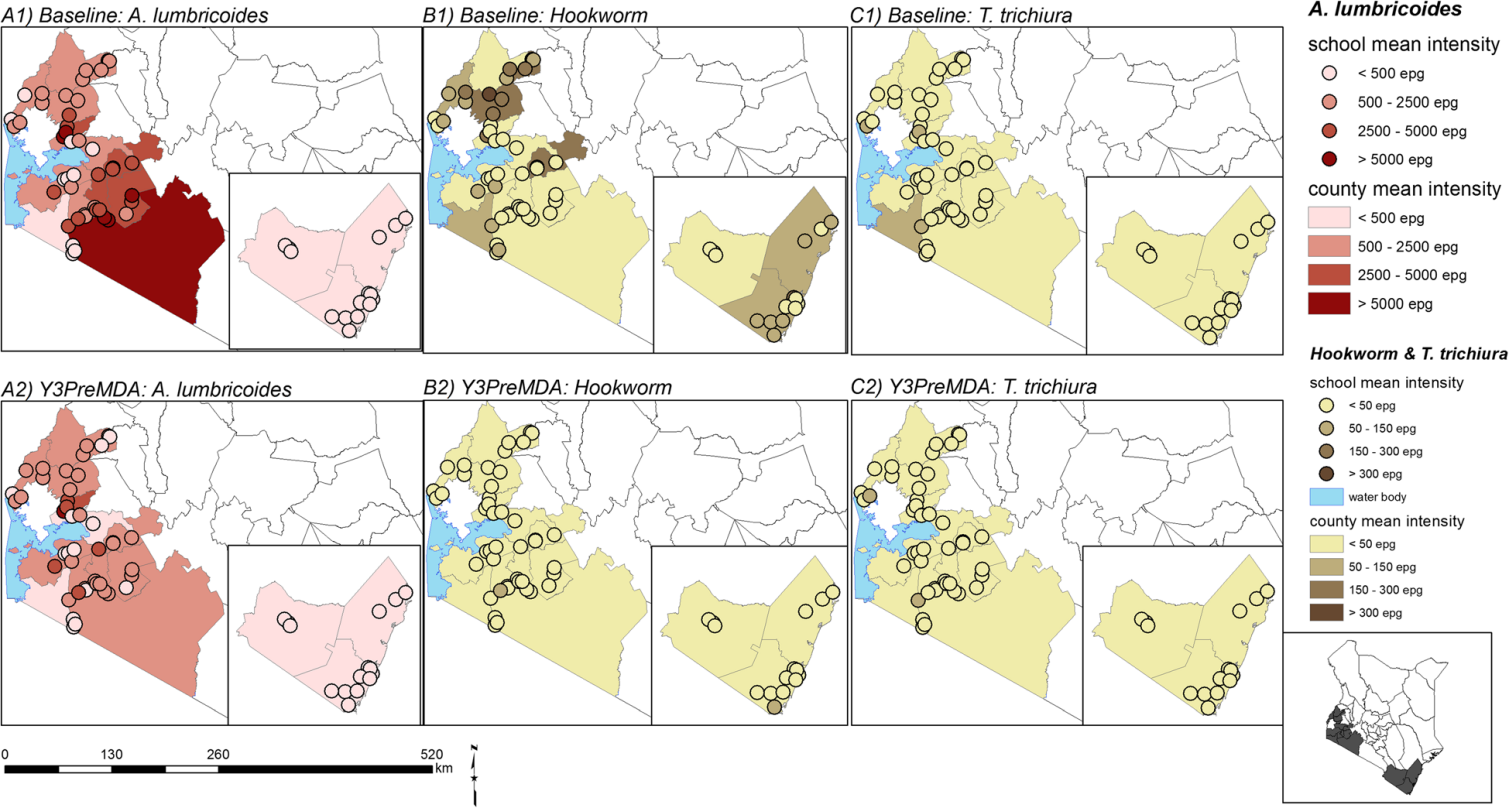
Observed geographical distribution of average infection intensity (epg)

**Fig. 3:**
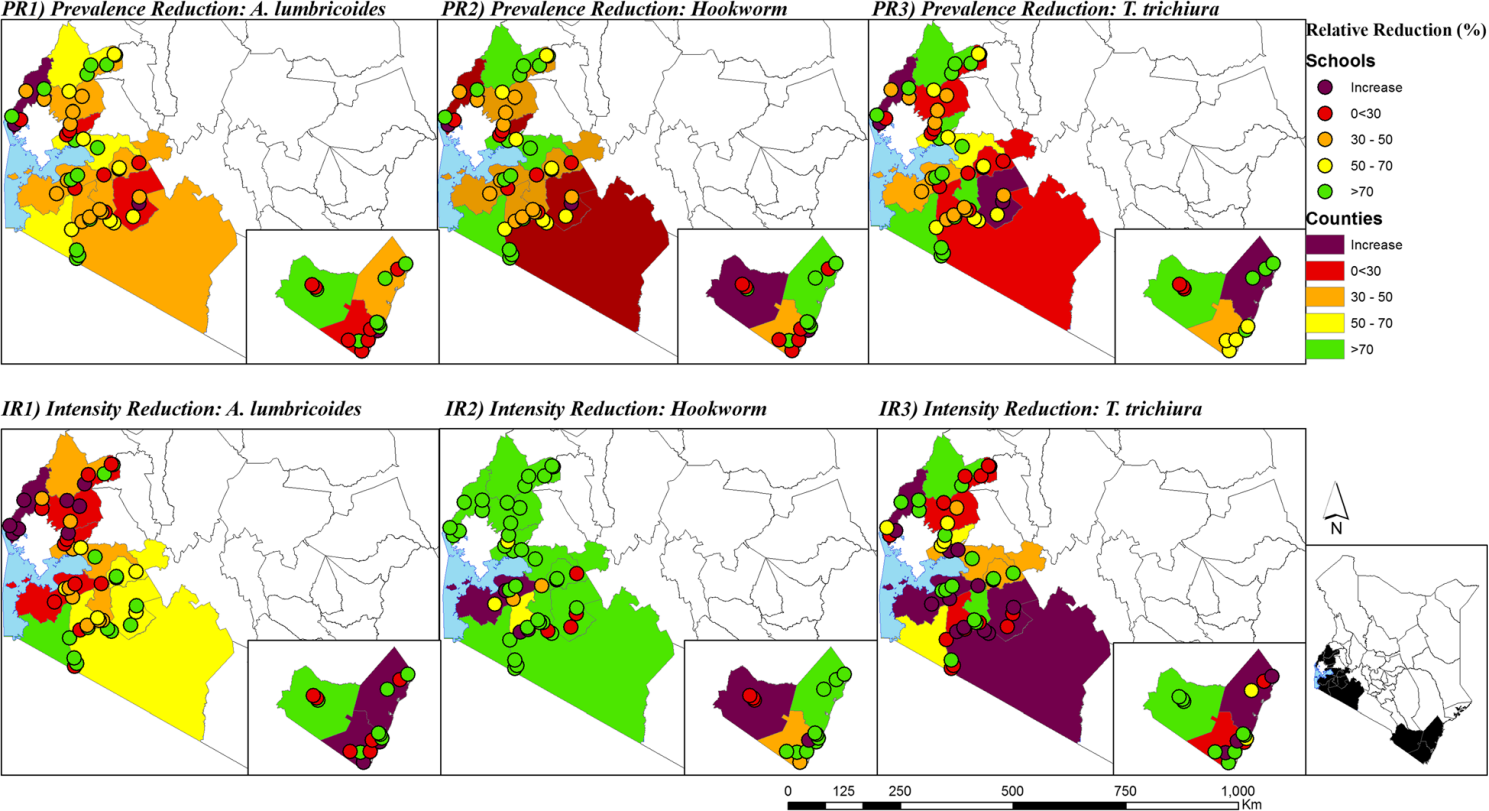
Infections relative reductions (%) in prevalence and average intensity in Y1 pre-MDA and Y3 pre-MDA surveys

Maps showing the observed geographical distribution of the combined STH prevalence, as well as the infection prevalence and intensity for each STH species in Y1 pre-MDA and Y3 pre-MDA are provided in Figs. 1 and 2. At Y1 pre-MDA, the observed prevalence and intensity of all STH species were highest in Western Kenya whereas in Coast, pockets of high prevalence of hookworms were observed. After the delivery of two rounds of MDA, a substantial reduction of hookworms and *T. trichiura* was observed across all the counties; Fig. 3 provides these relative reductions in prevalence and intensity after the two rounds of MDA.

### Reduction of moderate to heavy intensity infection from baseline to mid-term assessment

The prevalence of light, moderate and heavy intensity of infections from baseline to mid-term is summarised in Table 4. Examined STH infections were predominantly of light intensity. For STH combined, there was a significant reduction in both moderate and heavy intensity infections of 32.7 % (Wald test: *Z* = -6.47, *P* < 0.001) and 75.1 % (Wald test: *Z* = -2.20, *P* = 0.028), respectively. For hookworms, moderate intensity infections were significantly reduced by 86.9 % (Wald test: *Z* = -4.11, *P* < 0.001) and heavy intensity infections were non-significantly reduced by 61.7 % (Wald test: *Z* = -1.62, *P* < 0.105). For *A. lumbricoides*, reduction was 33.9 % (Wald test: *Z* = -6.79, *P* < 0.001) for moderate intensity infections, however, heavy intensity was not measured. Although, the light and heavy intensity infections with *T. trichiura* were significantly reduced by 34.6 % (Wald test: *Z* = -4.56, *P* < 0.001) and 94.0 % (Wald test: *Z* = -1.98, *P* = 0.047), respectively, its moderate intensity of infection non-significantly increased by 58.4 %.

**Table 4:** Prevalence of light, moderate and heavy mean intensity of infections [% (95 % CI)] in Y1 and Y3 and relative reductions % (Wald test: *Z*-statistic, *P*-value)

	Light	Moderate	Heavy
STH combined			
Y1 baseline	23.6 (22.0–25.4)	8.5 (7.2–10.1)	0.2 (0.1–0.5)
Y3 mid-term	10.6 (9.4–12.0)	5.7 (4.8–6.9)	0 (0–0.1)
Relative reduction (%)	55.1 (*Z* = -14.17, *P* < 0.001)	32.7 (*Z* = -6.47, *P* < 0.001)	75.1 (*Z* = -2.20, *P* = 0.028)
Hookworms			
Y1 baseline	15.1 (13.3–17.2)	0.2 (0.1–0.3)	0.1 (0–0.2)
Y3 mid-term	2.2 (1.7–3.0)	0 (0–0.1)	0 (0–0.1)
Relative reduction	85.2 (*Z* = -15.25, *P* < 0.001)	86.9 (*Z* = -4.11, *P* < 0.001)	61.7 (*Z* = -1.62, *P* = 0.105)
*A. lumbricoides*			
Y1 baseline	9.8 (8.7–11.2)	8.2 (6.9–9.8)	na^a^
Y3 mid-term	6.5 (5.6–7.5)	5.4 (4.5–6.5)	na^a^
Relative reduction	34.1 (*Z* = -6.87, *P* < 0.001)	33.9 (*Z* = -6.79, *P* < 0.001)	
*T. trichiura*			
Y1 baseline	6.4 (5.2–7.8)	0.2 (0.1–0.3)	0.1 (0–0.6)
Y3 mid-term	4.2 (3.1–5.5)	0.3 (0.2–0.6)	0
Relative reduction	34.6 (*Z* = -4.56, *P* < 0.001)	Increase (58.4 %) (*Z* = 1.70, *P* = 0.088)	94.0 (*Z* = -1.98, *P* = 0.047)

^a^ *A. lumbricoides* egg counts were truncated at 24,000 epg hence their heavy intensity of infection was not measured

### Patterns of annual treatment impact and re-infections

In this section we provide results from the 59 schools monitored yearly pre- and post- MDA delivery. Worm type specific prevalence and mean intensity of infection by survey is provided in Additional file 1: Table S4. Table 5 shows the relative reductions in prevalence and mean intensity of STH infections from Y1 to Y3 pre-MDA following two rounds of deworming. Absolute reductions in prevalence are also provided.

**Table 5:** Relative and absolute reduction, % (Wald test: *Z*-statistic, *P*-value), in prevalence and mean intensity of infection: Baseline to Y3 pre-MDA

Infection	Relative reduction (%)	Relative reduction (%)	Relative reduction (%)	Absolute reduction (%)
	Y1 to Y2	Y2 to Y3	Y1 to Y3	Y1 to Y3
STH combined				
Prevalence reduction	42.3 (*Z* = -7.33, *P* < 0.001)	14.3 (*Z* = -2.10, *P* = 0.035)	50.6 (*Z* = -9.02, *P* < 0.001)	16.8 (*Z* = -11.46, *P* < 0.001)
Intensity Reduction	36.6 (*Z* = -5.02, *P* < 0.001)	15.7 (*Z* = -1.45, *P* = 0.148)	46.6 (*Z* = -5.05, *P* < 0.001)	–
Hookworm				
Prevalence reduction	72.6 (*Z* = -6.37, *P* < 0.001)	45.8 (*Z* = -3.07, *P* = 0.002)	85.2 (*Z* = -8.15, *P* < 0.001)	14.0 (*Z* = -8.12, *P* < 0.001)
Intensity reduction	71.7 (*Z* = -3.60, *P* < 0.001)	66.3 (*Z* = -3.21, *P* = 0.001)	90.4 (*Z* = -6.36, *P* < 0.001)	–
*A. lumbricoides*				
Prevalence reduction	35.2 (*Z* = -6.06, *P* < 0.001)	1.0 (increase)^a^ (*Z* = 0.20, *P* = 0.843)	34.4 (*Z* = -6.17, *P* < 0.001)	6.7 (*Z* = -5.37, *P* < 0.001)
Intensity reduction	35.8 (*Z* = -4.66, *P* < 0.001)	14.5 (*Z* = -1.30, *P* = 0.192)	45.1 (*Z* = -4.66, *P* < 0.001)	–
*T. trichiura*				
Prevalence reduction	5.9 (*Z* = -0.43, *P* = 0.671)	40.5 (*Z* = -3.46, *P* = 0.001)	44.0 (*Z* = -3.86, *P* < 0.001)	2.4 (*Z* = -3.46, *P* < 0.001)
Intensity reduction	45.5 (increase)^a^ (*Z* = 1.35, *P* = 0.176)	44.3 (*Z* = -2.14, *P* = 0.033)	19.0 (*Z* = -1.06, *P* = 0.290)	–

^a^Indicates increase in prevalence or mean intensity rather than relative reduction

For hookworms and *A. lumbricoides,* higher reductions in prevalence and intensity of infections were observed after the first MDA than the second MDA. Specifically, after the first MDA, prevalence for STH combined were significantly reduced by 42.3 % with species-specific reductions of 72.6 % for hookworms, 35.2 % for *A. lumbricoides* and 5.9 % (non-significant) for *T. trichiura*. However, during the second MDA, the relative reductions for each species were all significant except *A. lumbricoides* which instead showed re-infection. The absolute reductions in infections after two rounds of MDA for all of the species were as follows: STH combined (16.8 %); hookworms (14.0 %); *A. lumbricoides* (6.7 %); and *T. trichiura* (2.4 %).

**Fig. 4:**
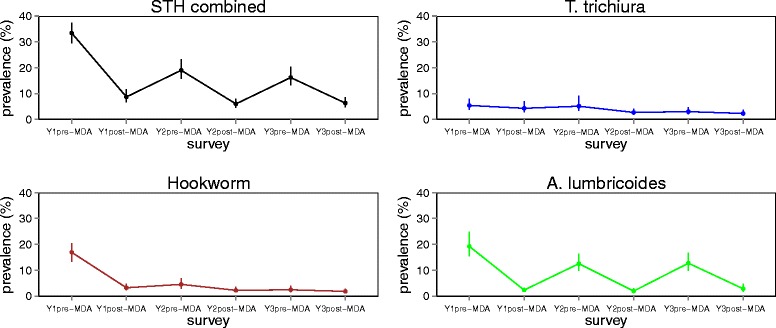
Prevalence (%) of STH infections from Y1 pre-MDA to Y3 post-MDA

**Fig. 5:**
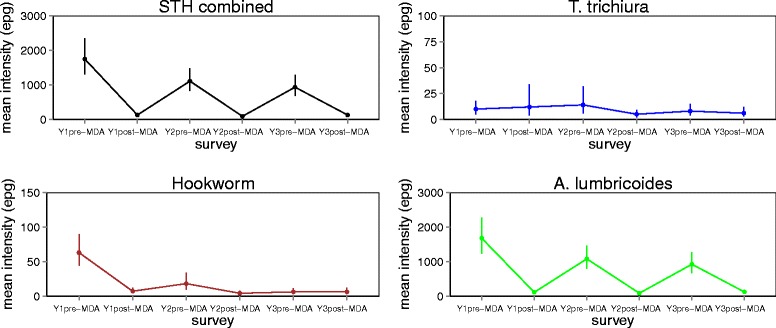
Average intensity (epg) of STH infections from Y1 pre-MDA to Y3 post-MDA

Table 6 provides the re-infection levels in prevalence and intensity of infections between different rounds of MDA. *A. lumbricoides* showed the highest re-infection in each of the treatment rounds (7.4 and 7.2 % after Y1 and Y2 treatment deliveries, respectively), followed by *T. trichiura* (4.7 and 2.8 % after Y1 and Y2 treatment deliveries, respectively) and hookworms (3.8 and 2.3 % after Y1 and Y2 treatment deliveries, respectively). Trends in STHs prevalence and intensity based on the 59 schools are shown in Figs. 4 and 5.

**Table 6:** Absolute increase, % (Wald test: *Z*-statistic, *P*-value), in prevalence (PI) and mean intensity of infection (II): Re-infections between different rounds of MDA

Infection	Y2 pre-MDA *vs* Y1 post-MDA	Y3 pre-MDA *vs* Y2 post-MDA	Y3 pre-MDA *vs* Y1 post-MDA
STH combined			
PI (%)	13.9 (*Z* = -19.40, *P* < 0.001)	11.1 (*Z* = -20.80, *P* < 001)	12.5 (*Z* = -19.58, *P* < 0.001)
II (epg)	616 (*Z* = 44.18, *P* < 0.001)	504 (*Z* = 38.96, *P* < 001)	524 (*Z* = 39.20, *P* < 0.001)
Hookworms			
PI (%)	3.8 (*Z* = -17.44, *P* < 0.001)	2.3 (*Z* = -16.62, *P* < 0.001)	2.8 (*Z* = -18.11, *P* < 0.001)
II (epg)	12 (*Z* = 9.32, *P* < 0.001)	5 (*Z* = 5.87, *P* < 0.001)	7 (*Z* = 8.13, *P* < 0.001)
*A. lumbrucoides*			
PI (%)	7.4 (*Z* = -19.63, *P* < 0.001)	7.2 (*Z* = -19.18, *P* < 0.001)	7.5 (*Z* = -19.19, *P* < 0.001)
II (epg)	590 (*Z* = 41.97, *P* < 0.001)	493 (*Z* = 38.26, *P* < 0.001)	507 (*Z* = 38.02, *P* < 0.001)
*T. trichiura*			
PI (%)	4.7 (*Z* = -13.60, *P* < 0.001)	2.8 (*Z* = -17.36, *P* < 0.001)	3.7 (*Z* = -15.20, *P* < 0.001)
II (epg)	13 (*Z* = 5.61, *P* < 0.001)	6 (*Z* = 6.46, *P* < 0.001)	10 (*Z* = 5.46, *P* < 0.001)

### STH infections hotspots

Hotspot analysis was performed to investigate if any school locations had exceptionally high STH infection levels (hotspots) during baseline and after two rounds of MDA (mid-term). During the baseline, 3/199 (1.5 %) schools (two from Homabay and one from Kisii counties), were identified as hotspots for hookworms, and 2/199 (1.0 %) schools, (both from Vihiga County), as hotspots for *T. trichiura* infection. No hotspots were reported for *A. lumbricoides* during baseline. After two rounds of MDA, only one school in Vihiga County was identified as hotspot for *T. trichiura* infection while no hotspots were found for hookworms and *A. lumbricoides* infections.

### Treatment coverage

**Fig. 6:**
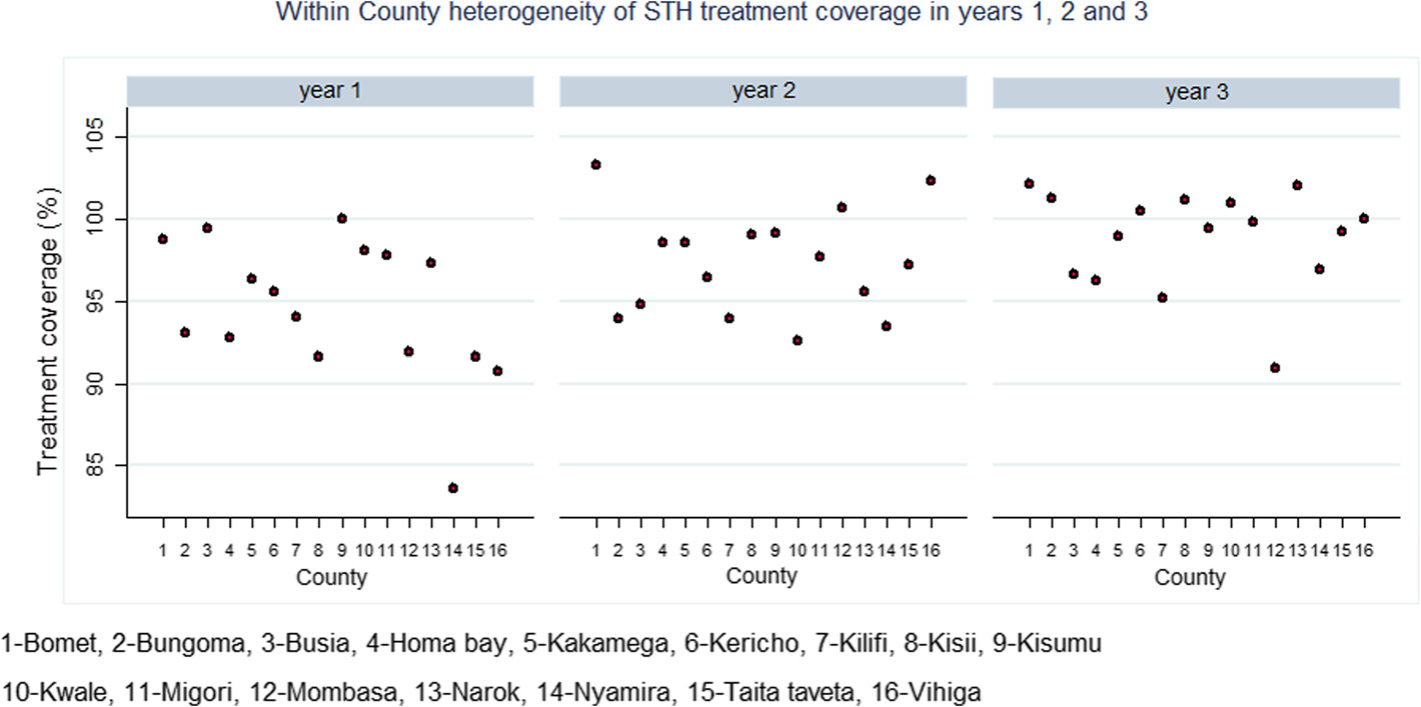
Median school treatment coverage (%) by county for Y1, Y2 and Y3

Deworming for STH has been carried out consistently over the three year period and all of the 16 counties included in the M&E programme were covered for treatment. Since 2012, totals of 5,056,530; 5,066,396; and 5,270,916 children (both enrolled and non-enrolled) have been dewormed for STH in the 16 counties in Y1, Y2 and Y3 respectively, with overall treatment coverage of 78.6, 76.0 and 82.2 % for the respective years. The deworming exercise has covered totals of 11,416; 12,521; and 13,585 primary schools for STH during Y1, Y2 and Y3 respectively. The school coverage was 94.7, 97.5 and 98.9 % for the respective years. The median school coverage per county is shown in Table 7 and Fig. 6.

**Table 7:** School treatment coverage (%) by county

County	Year 1		Year 2		Year 3	
	Total schools targeted	Schools treated (Coverage %)	Total schools targeted	Schools treated (Coverage %)	Total schools targeted	Schools treated (Coverage %)
Overall	12,060	11,416 (94.7)	12,843	12,521 (97.5)	13,740	13,585 (98.9)
Bomet	1005	992 (98.7)	851	879 (103.3)	935	955 (102.1)
Bungoma	1147	1067 (93.0)	1208	1135 (94.0)	1294	1310 (101.2)
Busia	559	556 (99.5)	598	567 (94.8)	654	632 (96.6)
Homa Bay	1264	1172 (92.7)	1381	1361 (98.6)	1430	1376 (96.2)
Kakamega	1107	1067 (96.3)	1215	1197 (98.5)	1380	1365 (98.9)
Kericho	587	561 (95.5)	808	779 (96.4)	858	862 (100.5)
Kilifi	718	675 (94.0)	758	712 (93.9)	829	789 (95.2)
Kisii	1415	1296 (91.6)	1361	1348 (99.0)	1411	1428 (101.2)
Kisumu	794	794 (100)	823	816 (99.1)	866	861 (99.4)
Kwale	475	466 (98.1)	487	451 (92.6)	506	511 (101.0)
Migori	1004	982 (97.8)	1107	1081 (97.7)	1152	1150 (99.8)
Mombasa	496	456 (91.9)	585	589 (100.7)	672	611 (90.9)
Narok	263	256 (97.3)	292	279 (95.5)	297	303 (102.0)
Nyamira	525	439 (83.6)	682	637 (93.4)	702	680 (96.9)
Taita Taveta	237	217 (91.6)	252	245 (97.2)	272	270 (99.3)
Vihiga	464	421 (90.7)	435	445 (102.3)	482	482 (100)

## Discussion

School-based deworming for the control of STH has been identified as one of the most cost-effective health interventions both in theory and practice [22–24]. Currently, an increasing number of STH endemic countries in the world, including Kenya [22], are implementing school-based deworming programmes and this paper presents the results of the impact of the first three years of the programme.

During baseline, observed prevalences were 32.3, 15.4, 18.1 and 6.7 %, with mean intenisties of 1756, 63, 1659 and 33 epg for STH combined, hookworms, *A. lumbricoides* and *T. trichiura*, respectively. Baseline STH combined prevalence was lower than those reported from other national school-based deworming programmes in Ethiopia (41 % [24]); Thailand (70 % [25]) and Ghana (36.3 % [26]). In this study, we found that *A. lumbricoides* was the most common STH followed by hookworms and *T. trichiura*. This reiterates evidence from previous reports around the world that suggest *A. lumbricoides* as being more prevalent compared to the other helminth infections [27, 28].

The prevalences of STH combined and each worm type after two rounds of treatment dropped to 16.4, 2.3, 11.9 and 4.5 % with mean intensities of 985, 8, 960 and 17 epg for STH combined, hookworms, *A. lumbricoides* and *T. trichiura*, respectively. Relative reductions of infections after two rounds of MDA were of; 50.6, 85.2, 34.4 and 44.0 %, respectively, for the prevalence, and 46.6, 90.4, 45.1 and 19.0 %, respectively, for the mean intensity. The reductions reported here are substantially higher than what was reported after seven years of deworming in Myanmar [29]. Further, our results corroborate other studies carried out in Kenya [16], which indicated a gradual decrease of observed combined STH prevalence over time across the country, with populations experiencing lower observed prevalence of infection in more recent years. The mid-term assessment showed that in overall the annual deworming is most effective against hookworms (reduction in intensity ≥ 90 %) but less effective against *A. lumbricoides* (reduction < 45 %) and *T. trichiura* (reduction < 44 %); a probable reflection of the higher rates of re-infection for *A. lumbricoides* and *T. trichiura*. The larger impact on hookworms has been highlighted previously in a review on STH infections in school-age children in sub-Saharan Africa [30].

The observed overall substantial decline in species-specific infections may be attributable to the direct impact of the school-based deworming programme, school health interventions, urbanisation and general improvements in socio-economic status, with associated improvements in living conditions, water, sanitation and hygienic (WASH) behaviour. For example, comparison of Demographic and Health Surveys from 1989 and 2009 reveals that access to an improved water source has risen nationally from 36.0 to 63.0 % of households [31]. Such secular changes are probably not unique to Kenya and it is likely that much of Africa has experienced a gradual decline in the prevalence of parasitic diseases, including helminth infections [16].

During the baseline, the observed prevalence and intensity of all STH species were highest in Western Kenya while in Coast pockets of high prevalence of hookworms were observed. In Y3 mid-term, there was substantial reduction of hookworms and *T. trichiura* across all counties. This finding agrees with a study by Pullan et al. [16], which spatially modeled the transmission of soil- transmitted helminths and showed that MDA was most warranted in the western and coastal areas of the country.

The prevalence and mean intensity of species-specific infections varied considerably by region, county and school levels. At baseline, Narok and Vihiga were the only counties which showed > 50 % prevalence of any STH. Relative reductions in prevalence showed that impact of MDA against *A. lumbricoides* was satisfactory (reduction ≥ 90 %) in only two counties, Mombasa and Taita Taveta, while it was satisfactorily effective for hookworms (reduction ≥ 90 %) in seven counties and for *T. trichiura* (reduction ≥ 90 %) in only one county. Further, at the individual level, patterns of infection and intensity varied by age-group and sex of the children.

Patterns of annual treatment impact and re-infections per school community were analysed in 59 schools using pre-post MDA surveys. The observed impact of the deworming programme between baseline and the mid-term assessment is determined by immediate reductions in infections after Y1 and Y2 treatment deliveries followed by re-infections between the treatments. Immediate infection reductions are likely to be influenced by the treatment coverage, which itself can be influenced by the broader context of the programme, such as quality of education and health systems, infrastructure and capacity to deliver health services and the economic situation. The rate of re-infection per school community is likely to be influenced by general risk factors for STH infections such as environmental conditions, WASH and socio-economic conditions and baseline infection levels [9].

In this study, we report re-infection rates of ≤ 8 % for each STH species after two rounds of MDA, with *A. lumbricoides* showing the highest rates of re-infection, followed by *T. trichiura* and hookworms. A recent meta-analysis showed that after MDA, the prevalence of STH infections rapidly recovers in most endemic areas: six months post-treatment, the prevalence of all three species reached or exceeded half the initial level; and at 12 months post-treatment follow-up, the prevalence of *A. lumbricoides* and *T. trichiura* usually returned to levels close to the initial pre-treatment, while levels of hookworm re-infection continued to fluctuate at about half pre-treatment level [13]. Rates of re-infections after treatment are influenced by species-specific characteristics, such as the life expectancy of the adult worm, the underlying intensity of transmission, environmental conditions that influence the development and survival of the free-living stages in the external environment, the relative rate of infection of different age groups, and drug efficacy [9, 32].

Notwithstanding the occurrence of re-infection, the first three years of intervention have been successful in reducing infections of moderate to heavy intensity, as well as the overall prevalence of STH infections. After the two rounds of MDA, infections of moderate intensity were significantly reduced by 32.7, 86.9 and 33.9 % for STH combined, hookworms and *A. lumbricoides*, respectively while they increased non-significantly for *T. trichiura* by 58.4 %. Other national control programmes have reported similar reductions, especially for any STH infections; particularly, after seven years of deworming in Myanmar, reduction of the infections of moderate to heavy intensity for any STH reduced from 18.5 % to less than 7 % [29]. In our study, we did not report cases of heavy infections for *A. lumbricoides* since their values were truncated at 24,000 epg; however, heavy infections for both hookworms and *T. trichiura* showed 0.1 % prevalence at baseline.

Lastly, this study points out the existence of STH transmission hotspots in the study area. During the baseline survey, three schools from Homabay and Kisii counties and two schools from Vihiga County were reported as transmission hotspots for hookworm and *T. trichiura* infections respectively but after two rounds of MDA, only one school was reported as hotspot for *T. trichiura* infection. These hotspot schools were only found in western Kenya, suggesting that the prevailing environmental conditions in the area, such as climate, soil moisture and relative atmospheric humidity, might favour transmission of these STH parasites. The substantial reduction of the number of detected hotspots after two rounds of MDA suggests the efficacy of albendazole.

We recognize that our study was not without limitations, the diagnosis was based on routine parasitological procedures and a single stool sample may underestimate the prevalence of helminth infection [33]. Moreover, the M&E programme does not follow-up individual children. Therefore, re-infections were estimated as the increase in infection levels by schools which may be influenced by variations in individual infections.

## Conclusion

Our study provides robust evidence for the impact of the Kenya national school-based deworming programme on STH infections. After three years of implementation, the programme is associated with reduction of moderate to heavy mean intensity of STH infection, but because of re-infection due to ongoing transmission, infection levels are yet to fall to very low levels. Additional intervention strategies may be required to interrupt transmission of STH. This study’s data can be useful in contextualizing the impact produced by other deworming programmes worldwide.

## Abbreviations

CIFF, Children’s Investment Fund Foundation; CIs, confidence intervals; ECD, early childhood education; KEMRI, Kenya medical research institute; M&E, monitoring and evaluation; MDA, mass drug administration; NTDs, neglected tropical diseases; ODK, open data kit; SBD, school-based deworming; STH, soil transmitted helminths; WASH, water, sanitation and hygiene; WHO, World Health Organization; Y1, year 1; Y2, year 2; Y3, year 3

## Additional files

Additional file 1: Figure S1. Outline of the 5-year monitoring and evaluation programme. **Table S1.** Intensity thresholds for light, moderate and heavy infections with *Ascaris lumbricoides*, *Trichuris trichiura*, hookworms and schistosomes. **Table S2.** Y1 baseline and Y3 mid-term prevalence % (95 % CI) and relative reduction (RR) by county. **Table S3.** Y1 baseline and Y3 mid-term average intensity (epg) (95 % CI) and relative reduction (RR) by county. **Table S4.** Overall prevalence (%) and average intensity (epg) of STH: Based on the 59 schools. (DOCX 54 kb) Additional file 2: School-level datasets from each of the survey rounds. Dataset1_02072016. (XLSX 84 kb)
