# Supplementary material for: Monitoring the impact of a national school based deworming programme on soil-transmitted helminths in Kenya: the first three years, 2012 – 2014
Source: Parasit Vectors. 2016 Jul 25;9:408. doi: 10.1186/s13071-016-1679-y (PMC4960809; doi:10.1186/s13071-016-1679-y)
Supplement: Additional file 1: Figure S1. — Outline of the 5-year monitoring and evaluation programme. Table S1. Intensity thresholds for light, moderate and heavy infections with Ascaris lumbricoides, Trichuris trichiura, hookworms and schistosomes. Table S2. Y1 baseline and Y3 mid-term prevalence % (95 % CI) and relative reduction (RR) by county. Table S3. Y1 baseline and Y3 mid-term average intensity (epg) (95 % CI) and relative reduction (RR) by county. Table S4. Overall prevalence (%) and average intensity (epg) of STH: Based on the 59 schools. (DOCX 54 kb) [file 13071_2016_1679_MOESM1_ESM.docx]

**Additional file 1**

**Additional file 1: Figure S1** Outline of the 5-year monitoring and evaluation programme

**Year 2 (2013)**

**Year 1 (2012)**

**Year 3 (2014)**

**Year 5 (2016)**

**Year 4 (2015)**

MDA

60 pre-post schools

60 pre-post schools

MDA

60 pre-post schools

60 pre-post schools

MDA

200 schools (including 60 pre-post schools)

60 pre-post schools

MDA

200 baseline schools (including 60 pre-post schools)

60 pre-post schools

MDA

200 schools (including 60 pre-post schools)

60 pre-post schools

**Additional file 1: Table S1** Intensity thresholds for light, moderate and heavy infections with *Ascaris lumbricoides*, *Trichuris trichiura*, hookworms and schistosomes

| **Helminth** | **Intensity Threshold** | | |
| --- | --- | --- | --- |
|  | **Light** | **Moderate** | **Heavy** |
| *A. lumbricoides* | 1 – 4999 epg | 5000 – 49999 epg | ≥ 50000 epg |
| *T. trichiura* | 1 – 999 epg | 1000 – 9999 epg | ≥ 10000 epg |
| Hookworms | 1 – 1999 epg | 2000 – 3999 epg | ≥ 4000 epg |
| *S. mansoni* | 1 – 99 epg | 100 – 399 epg | ≥ 400 epg |
| *S. haematobium* | 1 – 50 eggs/10ml urine |  | ≥ 50 eggs/10ml urine |

**Additional file 1: Table S2** Y1 baseline and Y3 mid-term prevalence % (95%CI) and relative reduction (RR) by county

| **County** | **Hookworms** | | | ***A. lumbricoides*** | | | ***T.* *trichiura*** | | | |  |
| --- | --- | --- | --- | --- | --- | --- | --- | --- | --- | --- | --- |
|  | **Y1  baseline** | **Y3  mid-term** | **RR**  **(%)** | **Y1  baseline** | **Y3  mid-term** | **RR**  **(%)** | **Y1  baseline** | **Y3  mid-term** | **RR**  **(%)** | |  |
| BOMET | 0.2 (0.0-0.6) | 0.1 (0.0-0.5) | 50.1 | 27.9 (18.9-41.3) | 20.9 (13.4-32.6) | 25.3* | 3.9 (2.1-7.3) | 5.7 (2.9-11.3) | | - | |
| BUNGOMA | 44.0 (36.4-53.2) | 1.8 (0.7-4.6) | 95.9* | 30.7 (21.8-43.1) | 9.7 (8.6-11.0) | 68.3* | 0.8 (0.4-1.6) | 0 | | 100* | |
| BUSIA | 20.9 (16.7-26.1) | 3.1 (1.9-5.0) | 85.1* | 14.4 (10.4-19.8) | 15.1 (11.8-19.3) | - | 12.5 (8.0-19.3) | 14.1 (7.9-25.0) | | - | |
| HOMA BAY | 14.7 (12.1-18.0) | 5.2 (3.4-7.9) | 64.8* | 17.3 (12.1-24.7) | 11.4 (7.1-18.2) | 34.1* | 5.8 (4.1-8.2) | 2.9 (2.0-4.4) | | 49.2* | |
| KAKAMEGA | 23.1 (17.5-30.6) | 0.8 (0.4-1.7) | 96.5* | 23.1 (18.0-29.7) | 15.0 (10.1-22.2) | 35.4* | 0.7 (0.3-1.7) | 0.7 (0.3-1.6) | | 3.3 | |
| KERICHO | 5.7 (2.9-11.1) | 0.1 (0.0-0.5) | 98.7* | 24.5 (16.9-35.6) | 14.6 (9.6-22.0) | 40.6* | 4.7 (2.6-8.7) | 4.0 (2.1-7.6) | | 15.1 | |
| KILIFI | 28.1 (21.8-36.4) | 1.4 (0.7-2.8) | 95.0* | 2.0 (1.0-4.2) | 0.4 (0.1-1.4) | 81.6* | 6.5 (3.7-11.5) | 1.4 (0.8-2.6) | | 78.4* | |
| KISII | 11.1 (6.9-17.8) | 1.4 (0.8-2.4) | 87.2* | 39.7 (32.0-49.1) | 25.4 (19.5-33.0) | 36.0* | 1.3 (0.7-2.3) | 1.1 (0.4-3.0) | | 15.6 | |
| KISUMU | 8.4 (5.5-12.9) | 0.5 (0.2-1.1) | 94.3* | 7.8 (5.1-12.0) | 2.4 (1.5-4.0) | 68.9* | 4.1 (2.0-8.3) | 2.0 (1.3-3.2) | | 50.4* | |
| KWALE | 27.7 (21.9-35.0) | 9.6 (6.0-15.5) | 65.3* | 0.8 (0.4-1.7) | 0.4 (0.2-1.0) | 45.1 | 8.9 (5.1-15.4) | 2.4 (1.1-5.4) | | 72.5* | |
| MIGORI | 20.1 (15.7-25.8) | 0.7 (0.4-1.3) | 96.5* | 3.4 (1.8-6.4) | 1.4 (0.7-2.7) | 58.6* | 0.7 (0.2-2.0) | 0.1 (0-0.8) | | 83.3* | |
| MOMBASA | 5.5 (2.1-14.5) | 0.8 (0.3-2.4) | 85.0* | 1.2 (0.7-2.1) | 0 | 100* | 15.6 (11.3-21.6) | 1.8 (1.0-3.3) | | 88.6* | |
| NAROK | 5.0 (2.3-10.9) | 0.8 (0.4-1.5) | 82.9* | 29.3 (20.2-42.3) | 20.3 (14.9-27.8) | 30.5* | 30.2 (20.9-43.5) | 26.6 (18.1-39.3) | | 11.7 | |
| NYAMIRA | 1.9 (0.9-4.2) | 0.4 (0.2-0.8) | 80.8* | 27.6 (19.0-40.0) | 18.8 (14.1-25.2) | 31.8* | 3.1 (0.6-16.7) | 0.5 (0.2-0.9) | | 84.7* | |
| TAITA | 0.9 (0.3-3.0) | 0.1(0.0-0.7) | 90.0 | 0.4 (0.1-1.7) | 0 | 100* | 1.4 (0.9-2.2) | 0.3 (0.1-1.1) | | 80.0* | |
| VIHIGA | 16.0 (9.3-27.6) | 1.8 (0.9-3.5) | 88.7* | 44.4 (36.9-53.4) | 33.9 (24.9-46.2) | 23.6* | 9.9 (5.0-19.5) | 7.2 (3.8-13.5) | | 83.9 | |
| **RR**; relative reduction in %,  * indicates a significant relative reduction (i.e p<0.05)  - indicates an increase in prevalence rather than relative reduction | | | | | | | | | | | |

**Additional fire 1: Table S3** Y1 baseline and Y3 mid-term average intensity (epg) (95%CI) and relative reduction (RR) by county

| **County** | **Hookworms** | | | ***A. lumbricoides*** | | | ***T.* *trichiura*** | | |
| --- | --- | --- | --- | --- | --- | --- | --- | --- | --- |
|  | **Y1  baseline** | **Y3  mid-term** | **RR**  **(%)** | **Y1  baseline** | **Y3  mid-term** | **RR**  **(%)** | **Y1  baseline** | **Y3  mid-term** | **RR**  **(%)** |
| BOMET | 0 | 0 | 83.4 | 3840(2519-5854) | 1488(800-2767) | 61.3* | 6(3-13) | 17(7-41) | - |
| BUNGOMA | 270(198-369) | 1(1-4) | 99.5* | 1566(1149-2135) | 813(588-1123) | 48.1* | 10(4-26) | 0 | 100* |
| BUSIA | 112(81-156) | 6(3-11) | 94.9* | 877(598-1285) | 1284(939-1757) | - | 33(19-59) | 59(23-152) | - |
| HOMA BAY | 27(18-40) | 30(10-94) | - | 1001(569-1761) | 798(450-1415) | 20.3* | 5(3-9) | 9(4-18) | - |
| KAKAMEGA | 129(87-192) | 1(0-2) | 99.4* | 1425(1036-1959) | 1156(721-1852) | 18.9 | 1(0-3) | 1(0-2) | 7.4 |
| KERICHO | 14(7-32) | 0 | 99.7* | 2738(1796-4173) | 1232(726-2090) | 55.0* | 18(7-47) | 11(3-42) | 41.5 |
| KILIFI | 47(35-63) | 3(1-9) | 93.3* | 28(8-100) | 4(1-27) | 85.4 | 6(3-15) | 3(1-16) | 46.8 |
| KISII | 23(10-53) | 11(3-40) | 53.9 | 5147(3560-7440) | 2180(1492-3185) | 57.6* | 1(0-2) | 1(0-2) | 28.2 |
| KISUMU | 15(8-29) | 0 | 97.6* | 423(171-1049) | 250(134-467) | 40.9 | 11(2-53) | 6(2-17) | 47.9 |
| KWALE | 117(50-271) | 28(15-53) | 75.8* | 15(4-58) | 35(9-132) | - | 15(5-48) | 6(2-16) | 59.7 |
| MIGORI | 19(10-36) | 1(0-2) | 95.8* | 131(63-273) | 38(7-199) | 70.7 | 0(0-1) | 0 | 66.6 |
| MOMBASA | 45(14-143) | 1(0-5) | 97.8* | 70(24-203) | 0 | 100* | 17(10-28) | 1(0-2) | 94.9* |
| NAROK | 44(9-213) | 2(0-8) | 95.6* | 3822(2503-5836) | 1539(930-2546) | 59.7* | 78(40-153) | 134(78-227) | - |
| NYAMIRA | 1(0-3) | 0 | 81.5* | 3031(1856-4951) | 1523(1088-2131) | 49.8* | 385(54-2730) | 3(1-15) | 99.2* |
| TAITA | 1(0-3) | 0 | 96.9* | 21(6-76) | 0 | 100* | 1(0-4) | 0 | 82.5* |
| VIHIGA | 103(55-195) | 11(4-31) | 89.7* | 3981(3103-5108) | 3191(2036-4999) | 19.9 | 31(11-82) | 13(5-34) | 57.2* |
| **RR**; relative reduction in %,  * indicates a significant relative reduction (i.e p<0.05)  - indicates an increase in intensity rather than relative reduction | | | | | | | | | |

**Additional file 1: Table S4** Overall prevalence (%) and average intensity (epg) of STH: Based on the 59 schools

| **Infection** | **Y1 baseline** | **Y1 post-MDA** | **Y2 pre-MDA** | **Y2 post-MDA** | **Y3 mid-term** | **Y3 post-MDA** |
| --- | --- | --- | --- | --- | --- | --- |
| **prevalence** | **% [95%CI]** | **% [95%CI]** | **% [95%CI]** | **% [95%CI]** | **% [95%CI]** | **% [95%CI]** |
| STH combined | 33.2 [29.4-37.4] | 8.8 [6.6-11.7] | 19.1 [15.7-23.3] | 6.0 [4.5-8.0] | 16.4 [13.2-20.3] | 6.4 [4.8-8.6] |
| Hookworms | 16.5 [13.3-20.4] | 3.2 [2.2-4.8] | 4.5 [2.9-6.9] | 2.2 [1.4-3.6] | 2.4 [1.5-3.9] | 1.8 [1.1-3.0] |
| A. *lumbricoides* | 19.5 [15.3-24.8] | 2.3 [1.6-3.2] | 12.6 [9.7-16.4] | 1.8 [1.1-3.0] | 12.8 [9.8-16.7] | 2.9 [1.7-4.8] |
| T. *trichiura* | 5.5 [3.8-7.9] | 4.4 [2.8-7.0] | 5.2 [3.3-8.1] | 2.7 [1.8-4.1] | 3.1 [2.0-4.8] | 2.3 [1.5-3.8] |
| **Mean intensity** | **epg [95%CI]** | **epg [95%CI]** | **epg [95%CI]** | **epg [95%CI]** | **epg [95%CI]** | **epg [95%CI]** |
| Hookworms | 63 [44-90] | 7 [4-12] | 18 [9-34] | 4 [2-8] | 6 [3-11] | 6 [3-12] |
| *A.* *lumbricoides* | 1678 [1234-2281] | 108 [68-171] | 1078 [797-1459] | 82 [48-138] | 921 [667-1273] | 119 [77-185] |
| *T.* *trichiura* | 10 [5-18] | 12 [4-34] | 14 [6-32] | 5 [2-9] | 8 [4-15] | 6 [3-12] |
|  | | | | | | |
